# Ganoapplanilactone C from *Ganoderma applanatum* Ameliorates Metabolic Dysfunction-Associated Steatotic Liver Disease via AMPK/mTOR-Mediated Lipid Regulation in Zebrafish

**DOI:** 10.3390/antiox14060637

**Published:** 2025-05-26

**Authors:** Yifan Guo, Mengke Zhang, Jiayang Xu, Mengyue Dong, Xin Chen, Anan Yang, Jinming Gao, Xia Yin

**Affiliations:** Shaanxi Key Laboratory of Natural Products & Chemical Biology, College of Chemistry & Pharmacy, Northwest A&F University, Yangling 712100, China

**Keywords:** *Ganoderma applanatum*, bioactive compounds, MASLD, metabolomics, AMPK/mTOR pathway

## Abstract

A phytochemical study of *Ganoderma applanatum* identified four predominant triterpenoids, with ganoapplanilactone C (GATC) exhibiting the most significant lipid-reducing effects in high-fat diet-fed zebrafish, surpassing atorvastatin at 5 μM. Histopathological analysis confirmed GATC’s protective effects on the liver against high-fat diet-induced damage. The Enzyme-Linked Immunosorbent Assay (ELISA) results showed a positive correlation between GATC treatment and liver health markers, as well as antioxidant enzymes, while they revealed a negative correlation with triglycerides and inflammatory cytokines. Metabolomic profiling demonstrates GATC’s impact on metabolites such as amino acids, fatty acids, and the mechanistic Target of Rapamycin (mTOR) signaling pathway, suggesting its role in regulating multiple metabolic processes. The increase in Adenosine Monophosphate-activated protein kinase (AMPK) phosphorylation in the GATC-treated groups indicates the activation of the AMPK/mTOR pathway, a key mechanism in lipid metabolism and liver protection. Molecular docking studies highlighted the importance of GATC’s spirocyclic ketone system and hydroxyl group in binding to target proteins. These findings underscore GATC’s potential as a therapeutic agent for metabolic dysfunction-associated steatotic liver disease (MASLD), emphasizing its superior efficacy compared to other triterpenoids due to its unique C-23 spiro 5/7 system. This study provides valuable insights into the prevention and treatment of MASLD using *G. applanatum*-derived compounds.

## 1. Introduction

Metabolic dysfunction-associated steatotic liver disease (MASLD), formerly known as nonalcoholic fatty liver disease (NAFLD), is a chronic metabolic disorder that has become the most prevalent liver disease globally, affecting individuals of all ages, including adults and children [1,2]. In 2024, an international consensus panel proposed the term “MASLD” to replace “NAFLD”, emphasizing the strong association of the disease with systemic metabolic dysregulation, such as obesity, insulin resistance, or type 2 diabetes, rather than the absence of alcohol consumption [3,4]. This updated nomenclature better reflects the pathophysiology of the disease and aligns with its multifactorial metabolic origins. MASLD is characterized by fat accumulation in the liver and encompasses a spectrum of liver conditions, ranging from simple fatty liver to more severe forms such as metabolic dysfunction-associated steatohepatitis (MASH), liver fibrosis, cirrhosis, liver failure, and hepatocellular carcinoma (HCC) [3,4,5]. Studies have elucidated the complex interplay of genetic, environmental, and lifestyle factors in the pathogenesis of MASLD. The pathogenesis of MASLD is initiated by excessive triglycerides in the liver, leading to lipotoxicity [6], insulin resistance [7], endoplasmic reticulum (ER) stress, and an inflammatory response [8], which can progress to cirrhosis and even liver cancer [9]. MASH, representing an advanced stage of MASLD, is characterized by the presence of hepatic steatosis, inflammation, ballooning degeneration, and/or fibrosis [10,11].

Lifestyle factors, particularly high-fat diets (HFDs), exhibit a complex and multifaceted relationship with the progression of MASH and MASLD. Mechanistically, the excessive consumption of HFDs may overwhelm the hepatic metabolic capacity, impairing cholesterol processing and storage mechanisms. This can lead to the accumulation of fat within liver cells, resulting in the formation of fat droplets in hepatocytes, which is a hallmark of MASLD [12]. Moreover, HFDs are associated with insulin resistance, a condition characterized by reduced cellular responsiveness to insulin. Insulin resistance is a key factor in the development of MASLD by promoting increased lipolysis in adipose tissue, resulting in elevated free fatty acid flux to the liver and subsequent hepatic lipid accumulation [13]. Furthermore, HFDs can induce low-grade inflammation, especially in the liver. Chronic inflammation is a key driver of MASLD progression, leading to liver cell injury, fibrosis, and, in some cases, cirrhosis. Additionally, HFDs can promote oxidative stress in the liver, damaging liver cells and exacerbating inflammation and fibrosis in MASLD. While HFDs are not the sole etiological factor in MASLD development, they significantly potentiate the underlying metabolic disturbances that drive disease pathogenesis and progression.

*Ganoderma*, known as Lingzhi or Reishi, represents a genus of medicinal mushroom that has been utilized for centuries in traditional Chinese medicine due to its potential therapeutic properties. This fungal is characterized by the presence of various bioactive compounds, including polysaccharides, ganoderma triterpenoids (GTs), and meroterpenoids. These compounds have demonstrated a range of biological activities, including antitumor, anti-inflammatory, antioxidant, antimicrobial, hypotensive, neuroprotective, hepatoprotective, nephroprotective, and antihyperlipidemic activities [14]. Among them, *G. applanatum*, a typical representative of the Ganoderma family, is widely consumed in Guangxi markets as a food material and has been used in folk medicine for treating a broad range of diseases, especially hyperlipemia. More importantly, special types of GTs with unique C-23 spiro 5/7 systems, including ganoapplanoid A and ganoapplanilactones A–C, have been reported to be found exclusively in this fungus. These compounds have been shown to promote lipid accumulation activity in adipocytes and exhibit anti-hepatic fibrosis activities in vitro [15,16]. Given the collective body of evidence, it is probable that C-23 spiro-type GTs possesses substantial efficacy in combating MASLD. In this study, comprehensive methods were used to evaluate the active substances related to the reversion of lipid accumulation and injury in an HFD zebrafish model. The activity-oriented separation method, non-targeted metabolomics, biochemical experiments, and molecular docking were employed. Based on the results, C-23 spiral lanostane GATC demonstrates superior performance, indicating a favorable outlook for its development.

## 2. Materials and Methods

### 2.1. Fungal Materials

The fruiting bodies of *G. applanatum* were collected from a forest in Baise city in Guangxi province in July 2022. The fungal specimen was taxonomically authenticated by Prof. Shuang-Tian Du from the College of Life Sciences. A voucher specimen of *G. applanatum* has been held in the College of Chemistry & Pharmacy, NWAFU, China. The 18 sRNA gene sequence is listed in the Supporting Information.

### 2.2. Activity-Guided Isolation of GTs

Powdered fruiting bodies of *G. applanatum* (50.0 kg) were extracted by refluxing with 75% EtOH (4 × 75 L × 2 h) at 65 °C. Then the extract was suspended in water, followed by liquid–liquid extraction with EtOAc to afford an EtOAc-soluble extract. The EtOAc extract (1.1 kg) was divided into seven parts (Fr. 1–Fr. 10) by using a silica gel chromatography column (CC, 300–400 mesh) eluted with a stepwise gradient of PE-EtOAc (100:0, 20:1, 4:1, and 1:1) and CH_2_Cl_2_-MeOH (20:1, 10:1, 5:1, and 0:100). All fragments were evaluated for their inhibitory effects on lipid accumulation at a concentration of 20 mg/mL on the HFD zebrafish model. Fr. 5 (5.6 g) showed the most potential activity and was further separated by reversed-phase C18 (RP-18) CC with gradient aqueous MeOH (MeOH–H_2_O, 20:80–100:0) to divide it into 10 sub-fractions (Fr. 5.1–Fr. 5.10). Fr. 5.1 to Fr. 5. 10 were then evaluated for the same activities, and Fr. 5.6 (451.0 mg) and Fr. 5.9 (886.0 mg) were then chosen for further separation. Ganoapplanilactone A (GATA, 243.7 mg) was purified followed by Sephadex LH-20 (CHCl_3_-MeOH, 1:1) and then recrystallized from Fr. 5.6. Additionally, ganoapplanilacone C (GATC, 99.6 mg) was isolated from Fr. 5.6 followed by Sephadex LH-20 (CHCl_3_-MeOH, 1:1), Sephadex LH-20 (Actone, Tokyo, Japan), and then semi-HPLC with MeOH-CH_3_CN (60:40, *v*/*v*; r. t. 20 min). Fr. 5.9 was separated by a silica gel chromatography column to obtain eight subfractions. Fr. 5.9.6 was subjected to semi-HPLC with MeOH-H_2_O (50:50, *v*/*v*, r. t. 18 min) as the eluent to yield methyl ganosate I, (MGI, 357.3 mg, content). Fr. 5.9.8 was separated by semi-HPLC CH_3_CN-H_2_O (45:55, *v*/*v*, 16 min) to yield ganoderenic acid G (GAG, 129.4 mg).

### 2.3. Animal Ethics and Welfare

All study protocols were approved by the Laboratory Animal Center of Northwest A&F University (Yangling, China) under the approval number: SYXK (Shan)2022-03. This study was conducted according to the European Community guidelines (EEC Directive of 1986; 86/609/EEC).

### 2.4. Breeding and Spawning of Zebrafish

Wild-type AB strain zebrafish were maintained under standard conditions, in accordance with previous methods [17]. Adult AB strain wild-type zebrafish were kept in E3 water at 28 °C with a pH range of 6.9 to 7.2, conductivity of 450–500 mS-cm^−1^, and hardness of 53.7–71.6 mg/L CaCO_3_, under a standard light/dark cycle of 14 h of light and 10 h of darkness. The zebrafish were fed live Artemia shrimp larvae three times a day. Each mating pair, consisting of a male-to-female ratio of 3:2, typically produces about 200 to 300 embryos. These embryos were incubated at 28 °C in clean E3 water.

### 2.5. HFD Modeling and Experimental Grouping

In the HFD model, two distinct age groups of zebrafish were employed to achieve the specific experimental objectives. The first group consisted of zebrafish larvae at 5 days post-fertilization (5 d.p.f.), which were utilized for Oil Red O staining (Section 2.6), liver functional index (Section 2.8), triglyceride (TG) levels (Section 2.9), assessment of oxidative stress (Section 2.10), and untargeted metabolomics analysis (Section 2.12). Zebrafish larvae were selected due to their inherent advantages, including optical transparency, high reproductive efficiency, and ease of maintenance, which not only ensure the reliability of experimental outcomes but also reduce the quantity of test samples required. The second group involved 6-month-old adult zebrafish, from which liver tissues were excised and processed into histological sections. These sections were subsequently analyzed to evaluate the extent of tissue damage induced by the experimental conditions (Section 2.7), as well as the potential therapeutic effects of the test compounds in mitigating liver injury and inflammation, such as ELISAs to measure the expression of IL-1β/6 and TNF-α (Section 2.11) and the expression of p-AMPK and AMPK (Section 2.13). This dual-age approach allows for a comprehensive investigation of both metabolic and histological alterations associated with the HFD model. To establish MASLD models, zebrafish larvae (5 d.p.f.) and 6-month-old zebrafish were administered a 0.5% egg yolk powder. Larvae were distributed into 24-well plates, with up to 15 per well. Each well received 2 mL of 0.5% HFD solution. Post 48 h of modeling, larvae underwent Oil Red O staining or treatment administration for an additional 24 h [17,18,19]. Atorvastatin, a common lipid-lowering agent, was used as a positive control [20]. In detail, zebrafish larvae at 5 d.p.f. were administered 0.5% egg yolk powder solution three times daily (1 h exposure per session), with intervals of at least 5 h between administrations. The disease model was successfully established 48 h post-treatment. For 6-month-old adult zebrafish, the identical protocol was implemented, followed by subsequent experiments after 48 h of model establishment. In accordance with humane principles and animal welfare guidelines, zebrafish in this study were euthanized using hypothermic shock. Larvae were collected into centrifuge tubes and immediately subjected to rapid freezing in liquid nitrogen. Adult zebrafish were immersed in an ice-water slurry for hypothermic anesthesia, followed by dissection after confirmation of complete cessation of gill movement. The study design encompassed a blank control, a model group, and four treatment groups with different test compounds with concentrations of 2.5 μM, 5 μM, 10 μM, and 20 μM, respectively. Both atorvastatin and the test compounds were dissolved in DMSO, with the maxi-mum DMSO concentration not exceeding 0.1%. The control group received 0.1% DMSO only.

### 2.6. Oil Red O Staining

Following the completion of the experimental treatments, zebrafish were fixed with 4% paraformaldehyde solution for 4 h and then dehydrated in 60% propylene glycol solution for 15 min at room temperature. The samples were then stained with Oil Red O while protecting from light for 3–4 h and imaged using a Nikon stereotactic fluorescence microscope. Lipid deposition in vascular, hepatic, and intestinal tissues was visualized, and the staining intensity was quantified using ImageJ software (https://imagej.net/ij/index.html, accessed on 25 July 2022) to determine the mean gray value, indicative of the relative lipid content [21].

*Lipid clearance* in zebrafish could be quantified using the mean gray value from images as shown in the following formula.

$$Lipid\;clearance\left(\%\right)=\frac{Vdosing-Vmodel}{Vmodel}\times 100\%$$

### 2.7. Histopathological Analysis

Liver tissue samples were collected from 6-month-old zebrafish for histological analysis. Following euthanasia, the fish were immediately dissected, and liver tissues were carefully excised and immersed in 4% paraformaldehyde (PFA) fixative solution at 4 °C for 24–48 h to ensure optimal tissue preservation. The fixed tissues were then processed through a standard histological protocol by Servicebio Co., Ltd. (Wuhan, China), which included tissue dehydration, paraffin embedding, sectioning (4–5 μm thickness), and staining procedures. For histological examination, tissue sections were stained with hematoxylin and eosin (H&E) for general morphological assessment.

The stained sections were examined using a LECIA DM6 B automatic fluorescence microscope (Leica Microsystems Inc., Wetzlar, Germany) equipped with bright field filter sets and an MC190HD camera (Leica Microsystems Inc., Wetzlar, Germany) with high resolution.

### 2.8. Analysis of Liver Function Index

Liver function assessment was performed according to the manufacturer’s protocol using commercial assay kits. Zebrafish larvae (*n* = 30 per group) were washed three times with PBS, blotted dry, and weighed. The tissues were then homogenized in PBS at a weight-to-volume ratio of 1:9 (g/mL). The homogenate was centrifuged at 4 °C for 10 min at 5000 rpm to separate the supernatant. The supernatant samples were analyzed for aspartate aminotransferase (AST) and alanine aminotransferase (ALT) using assay kits. After a 15 min incubation at room temperature, the absorbance was measured at 510 nm for AST and 505 nm for ALT using a microplate reader (BioTek, Winooski, VT, USA). Each dosing group contributed triplicate samples, resulting in a total of 90 juvenile fish for analysis.

### 2.9. Detection of Total Triglyceride Content

In accordance with the kit guidelines, zebrafish larvae were washed three times to remove residual water. The tissues were then carefully blotted dry and weighed. Anhydrous ethanol was added to the tissues at a weight-to-volume ratio of 1:9 (g/mL) for homogenization. This mixture was centrifuged at 4 °C for 10 min at 5000 rpm to separate the supernatant. The total triglyceride (TG) content in the supernatant was determined using a triglyceride assay kit and measured with a microplate reader at an absorbance wavelength of 500 nm.

### 2.10. Measurement of Oxidation Level

After precise weighing, the juvenile fish tissues were homogenized with the SOD sample preparation solution (1:9 g/mL) provided in the kit. The homogenate was centrifuged at 4 °C for 10 min to obtain the supernatant. The SOD assay working solution was prepared as per the kit’s instructions and incubated at 37 °C for 30 min. Total SOD activity was then measured at 450 nm using a microplate reader. The sample preparation method for the CAT, MDA, and GSH-PX activity assays varied slightly from that of the SOD. Instead of the extraction medium, PBS was used. After collecting the supernatant, the protein concentration was determined using the BCA Protein Concentration Determination Kit (CAT Activity Detection Kit, measured at 405 nm; MDA content at 532 nm; and GSH-PX activity at 412 nm) using a microplate reader.

### 2.11. Measurement of Inflammatory Levels

Given the small size of the 6-month-old zebrafish liver, six fish were used per group. Livers from two fish were pooled in each centrifuge tube, resulting in three biological replicates per group. Samples were snap-frozen in liquid nitrogen and stored at −80 °C. Each liver sample in the centrifuge tubes was accurately weighed, and the extraction was performed following the standard protocol for tissue sample extraction, with a material-to-liquid ratio of 1:9 (g/mL). During the actual operation, an appropriate dilution was carried out according to the kit’s instructions. ELISA kits for IL-1β, IL-6, and TNF-α were used to assess the level of inflammation. Additionally, the detection conditions using a microplate reader were standardized, with all assays set to an absorbance wavelength of 450 nm.

### 2.12. Non-Targeted Metabolomics

Zebrafish larvae (5 d.p.f.; *n* = 50; 350 larvae per dosing group in septuplicate) were exposed to 0.5% egg yolk powder for 48 h for modeling. After modeling and GATC administration, tissues were immediately flash-frozen in liquid nitrogen, stored at −80 °C overnight, and subsequently lyophilized. The lyophilized tissues were accurately weighed and extracted with 80% methanol, homogenized, and centrifuged. The supernatant was transferred to liquid phase vials. Post-extraction, the tissue was sent to Shanghai BioTree Technology Co. for the non-targeted metabolomics assay. The data were analyzed by MetaboAnalyst 5.0. Initially, orthogonal-partial least squares discriminant analysis (OPLS-DA) was performed for each group of metabolites. The peak intensities of the metabolites were analyzed with a *p*-value < 0.05 as the threshold, and the differentially expressed metabolites (DEMs) of each group are displayed in a heatmap (Supporting Information) [22].

### 2.13. Measurement of p-AMPK and AMPK

Sample were prepared similarly to those for measuring inflammatory levels. A 10 μL aliquot of extraction solution was added, followed by 40 μL of sample dilution solution during the spiking process, achieving a 5-fold dilution. ELISA kits were used in this study to measure the expression of *p*-AMPK and AMPK, with the absorbance recorded at 450 nm using a microplate reader.

### 2.14. Molecular Docking

We conducted molecular docking using AutoDockTools 1.5.7 [23] and performed protein pre-processing and post-docking visualization with PyMol [24]. The PDB format file of the protein AMPKα2 (PDB ID: 4CFF) was downloaded from the PDB database. Water molecules and the ligand were removed, and the files were renamed and saved in pdbqt and prepared for docking in AutoDock. Ligands were imported into AutoDock’s Ligand module and pre-processed using the Torsion Tree. The Grid operation was performed to set the appropriate docking range (Grid box) for both proteins and small molecules. After setup, AutoGrid was run, and the program was allowed to complete. Once the Grid was finished, docking was performed, and the results were analyzed based on binding energy, with strong binding indicated by values < −7.0 kcal/mol. The interactions and hydrogen bonds between small molecules and target proteins were visualized using PyMol.

### 2.15. Data Analysis

For data analysis, graphs were generated using GraphPad Prism 8.0.2. Data are presented as the mean ± standard error of the mean (Mean ± SEM). Statistical analyses were performed using *t*-tests and one-way ANOVA to compare differences between groups. Normality and Lognormality tests were performed using the Shapiro–Wilk test. The results are presented as the mean ± standard error of the mean (mean ± SEM) with ns, *p* > 0.05, *p* ≤ 0.05, *p* ≤ 0.01, *p* ≤ 0.001, and *p* ≤ 0.0001. Analyses were performed using GraphPad Prism software 8.0.2 (GraphPad Software Inc., San Diego, CA, USA).

## 3. Results

### 3.1. The Effect of GTs on HFD-Induced Lipid Accumulation in Zebrafish

**Figure 1:**
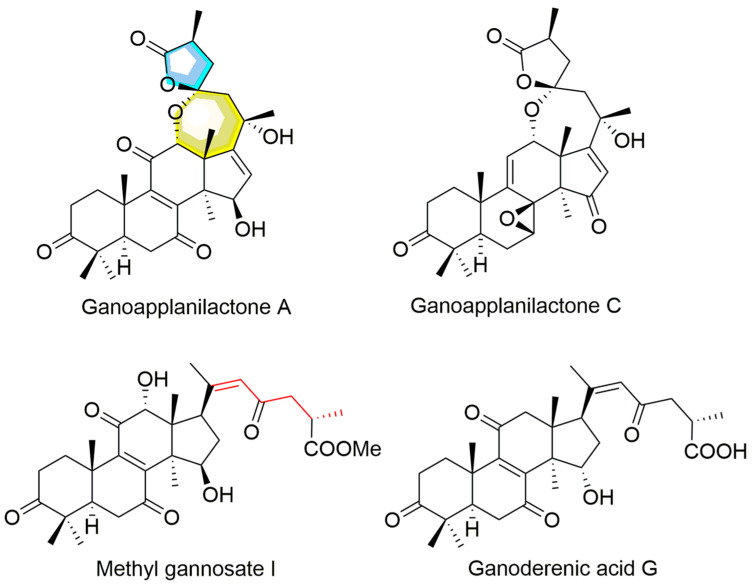
The structures of ganoderma triterpenoids from *G. applanatum*.

**Figure 2:**
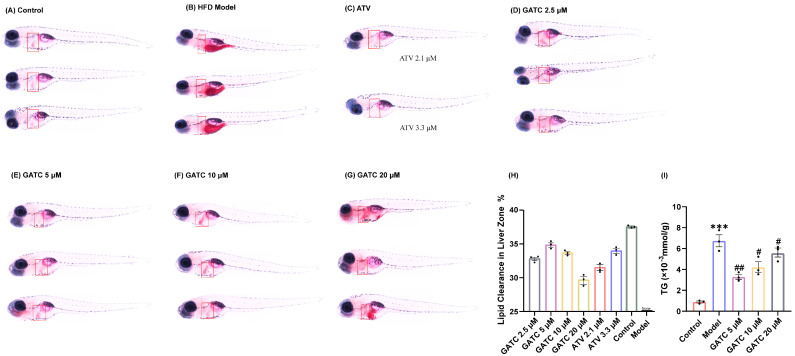
Oil Red O staining and determination of triglyceride content. The liver is marked in the red square. (**A**) Control group staining results. (**B**) 0.5% HFD model group staining results. (**C**) Positive control (ATV) group staining results. (**D**) GATC 2.5 μM group staining results. (**E**) GATC 5 μM group staining results. (**F**) GATC 10 μM group staining results. (**G**) GATC 20 μM group staining results. (**H**) Semi-quantitative analysis of lipid clearance in the liver zone. (**I**) Determination of triglyceride content in zebrafish (Danio rerio). (vs. Control group *** *p* < 0.001; vs. Model group ## *p* < 0.01; # *p* < 0.05, *n* = 3).

Under the guidance of hypolipidemic activity, four ganoderma triterpenoids (GTs) were isolated. Their structures were determined by NMR and confirmed through literature comparison (Supporting Information), and they were ultimately identified as ganoapplanilactone A (GATA), ganoapplanilactone C (GATC), methyl gannosate I (MGI), and ganoderenic acid G (GAG) (Figure 1). The GTs were tested for their exhibition of lipid accumulation. Zebrafish larvae (5 d.p.f.) were exposed to HFDs for 48 h, and whole-mount Oil Red O staining revealed severe fat deposition compared to the control group. This effect was reversible by atorvastatin (ATV) treatment (Figure 2A–C). Statistical analysis using ImageJ software indicated that both the grayscale value and the fluorescence intensity of the liver tissue in the experimental model group were significantly higher than those in the control group. At a concentration of 10 μM, all GTs exhibited lipid-lowering activity, with lipid clearance rates of 9.47% for MGI, 16.36% for GAG, 24.98% for GATA, and 33.74% for GATC. Among them, GATC showed the most promise and was selected for subsequent in vivo and in vitro experiments. In the GATC treatment groups, concentrations of 2.5, 5, 10, and 20 μM all significantly reduced lipid accumulation (Figure 2D–G). Calculation of the lipid clearance rate in the liver region showed that 2.5 μM GATC had lipid clearance activity comparable to 2.1 μM ATV, with the best effect observed at 5 μM, surpassing the positive control (Figure 2H). An increase in the GATC concentration led to a decrease in lipid clearance activity. Therefore, 5 μM and 10 μM were determined to be the optimal dosages of GATC for improving lipid accumulation in the zebrafish model. The total triglyceride (TG) content measurements were consistent with the trends in lipid clearance activity (Figure 2I).

### 3.2. The Effect of GATC on Liver Injury

**Figure 3:**
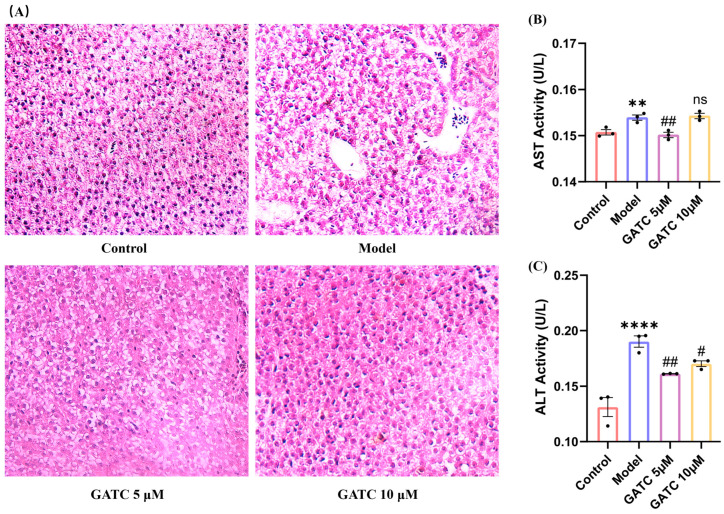
Histopathologic sections of zebrafish liver and biochemical indices of liver function. (**A**) Liver sections from different groups (20× magnification). (**B**) ALT activity in zebrafish tissues. (**C**) AST activity in zebrafish tissues.. (vs. control group **** *p* < 0.0001, ** *p* < 0.01; vs. model group ## *p* < 0.01, # *p* < 0.05; ns, non-significant > 0.05, *n* = 3).

As shown in Figure 3, histological alteration visualized by H&E staining confirmed the protective effect of GATC against HFD-induced liver damage. The livers in the control group exhibited a normal arrangement of hepatocytes with no signs of disease, whereas the model (HFD) group presented significant pathological alterations. These changes included an increase in the number of medium-sized and small lipid droplets, disrupted hepatocyte distribution, inflammatory cell infiltration, and an anomalous aggregation of red blood cells within the hepatic sinusoids (Figure 3A). Treatment with GATC led to a gradual improvement in these hepatic pathological alterations. Furthermore, compared to the control group, the model group displayed an increase in ALT and AST levels, indicating liver damage by HFDs. GATC at 5 μM significantly reduced these indicators (Figure 3B,C). The 20 µM treatment group showed similar improvements to the 10 µM group, but neither was as effective as the 5 µM group, further confirming that the 5 µM group is the most effective in the zebrafish model. In summary, these data suggested that GATC treatment can protect the livers from HFD-induced damage to a certain extent.

### 3.3. The Effect of GATC on Antioxidant Capacity

**Figure 4:**
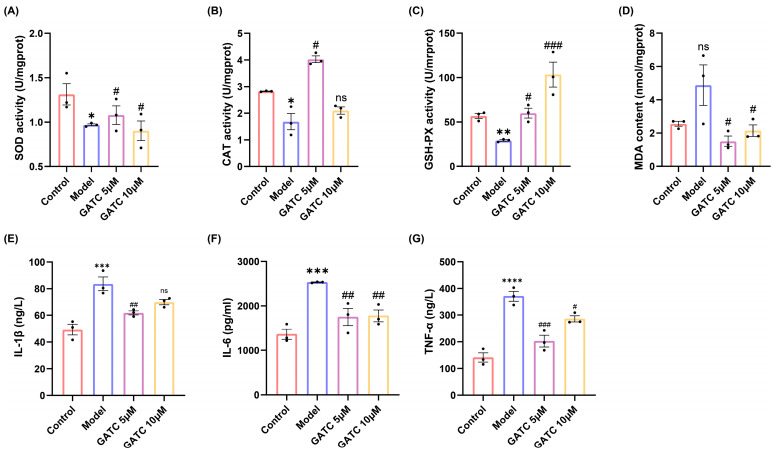
Biochemical indicators of oxidative stress and inflammation. (**A**) SOD activity. (**B**) CAT activity. (**C**) GSH-PX activity. (**D**) MDA content. (**E**) IL-1β expression. (**F**) IL-6 expression. (**G**) TNF-α expression. (vs. control group **** *p* < 0.0001, *** *p* < 0.001, ** *p* < 0.01, * *p* < 0.05; vs. model group ### *p* < 0.001, ## *p* < 0.01, # *p* < 0.05, n = 3).

In light of the findings from the initial trio of experiments, which established 5 µM and 10 µM as the optimal concentrations for GATC dosing, these concentrations were systematically employed in subsequent in vitro and in vivo studies. Consumption of HFDs led to a significant reduction in the levels of key antioxidant enzymes, including superoxide dismutase (SOD), catalase (CAT), and glutathione peroxidase (GSH-Px), as well as a decrease in their scavenging capacity. Concurrently, HFD consumption resulted in an increase in the malondialdehyde (MDA) content, a biomarker of oxidative stress, compared to the control group. Conversely, the administration of GATC effectively attenuated these HFD-induced oxidative stress parameters, highlighting the antioxidant potential of GATC in mitigating the HFD-induced alterations in fish (*p* < 0.05, Figure 4A–D).

### 3.4. The Effect of GATC on Acute Phase Inflammatory Inducers

The levels of IL-6, TNF, and IL-1β were significantly increased in the model group compared to the control group, indicating the overproduction of inflammatory cytokines secreted by hepatocytes due to excessive HFD intake. Ultimately, the alterations observed were significantly ameliorated by GATC treatment. These findings suggest that GATC intervention mitigates inflammatory responses, thereby conferring hepatoprotective effects (Figure 4E–G).

### 3.5. The Effect of GATC on Metabolic Profiles with Different Concentrations

**Figure 5:**
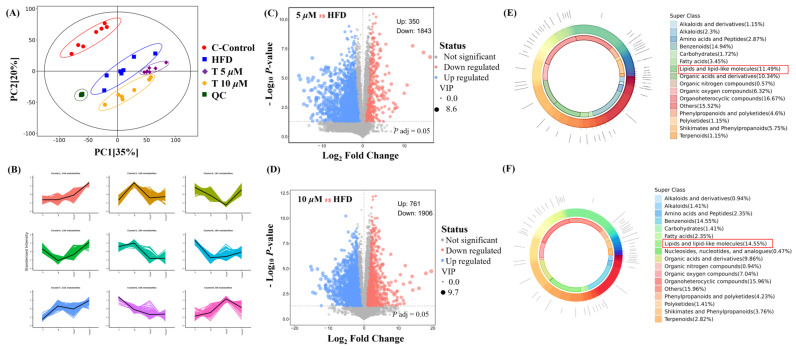
Metabolomics analysis. (**A**) PCA plot of control, HFD, 5 μM GATC, and 10 μM GATC; (**B**) K-Means of control, HFD, 5 μM GATC, and 10 μM GATC; (**C**,**D**) Volcano plot showing the differentially regulated genes by GATC and HFD; (**E**,**F**) Chemical classification of metabolites of 5 μM and 10 μM. (QC, quality control indicates that the method has good stability and reproducibility, and the data obtained are reliable; The Variable Importance in Projection (VIP) value is usually calculated when using OPLS-DA to measure the contribution of each variable (in this case, metabolites) to the model’s classification or predictive ability; the higher the VIP value, the more important the metabolite is in distinguishing between different groups).

Untargeted metabolomics was employed to comprehensively reveal the differences among the control (C), model (M), and GATC treatment groups (5 T—5 μM and 10 T—10 μM). Principal component analysis (PCA) was used to analyze the untargeted metabolomics data. As depicted in Figure 5, significant disparities were observed in the coordinate values on both the X and Y axes when comparing the control group to the model group, as well as between the 5 T and 10 T groups. These findings indicated substantial differences in the overall metabolic profiles across the C, M, 5 T, and 10 T groups, with a 95% confidence interval. To visualize the group separation and identify significantly different metabolites (DEMs), an orthogonal partial least squares–discriminant analysis (OPLS-DA) model was employed. Subsequently, a 7-fold cross-validation was conducted to ascertain the model’s predictive accuracy by calculating the R2 and Q2 values. R2 demonstrates the extent to which the variation in a variable is explained, while Q2 explained how well a variable could be predicted (Figure 5A). To elucidate the trends in relative content changes of metabolites across different groups, the relative contents of all differential metabolites identified by standard methods in all group comparisons were subjected to z-score normalization. This preprocessing step was followed by k-means clustering analysis to categorize the metabolites based on their expression patterns (Figure 5B). Metabolites were considered as DEMs if they exhibited a *p*-value of less than 0.05 and VIP value of more than 1.0 between the M and 5 T groups, according to the metabolomics results. Comprehensive details regarding differential metabolites are provided in the Table S1, in Supplementary Materials. Among these, 350 metabolites were upregulated and 1842 metabolites were downregulated in the 5 T group compared to those in the M group (Figure 5C). In the 10 T group, 761 metabolites were upregulated and 1906 metabolites were downregulated (Figure 5D). The majority of DEMs were categorized under groups that encompass organ heterocyclic compounds, lipids and lipid-like components, benzene and its substituted derivatives, amino acid and their derivatives, alkaloids and their derivatives, and nucleotides and their derivatives, among others. Additionally, GATC increased the relative abundance of asparagine, myo-inositol, CDP-choline, and 2-hydroxyethanesulfonic acid and decreased N2-acetylornithine and glycose in the 5 T group (Figure 5E). Subsequently, in the 10 T group, GATC increased 3-methoxytyramine, asparagine, creatine, CDP-choline, glutamine, and myo-inositol (Figure 5F).

**Figure 6:**
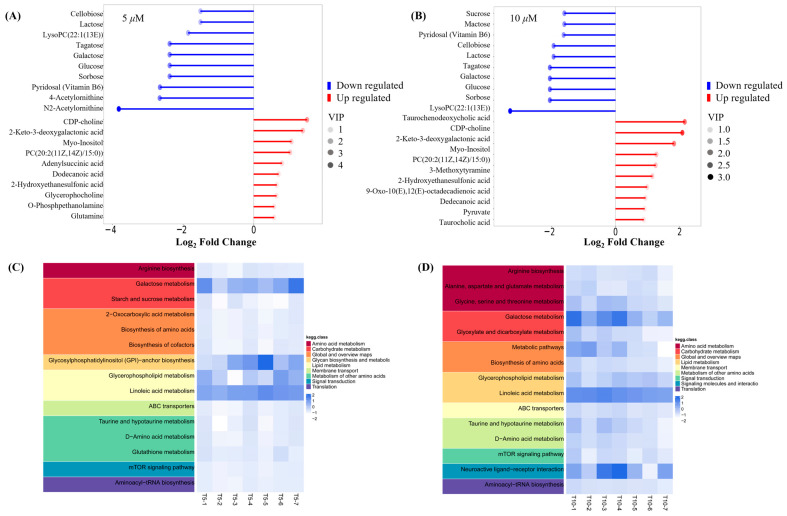
Metabolite trend analysis and bioinformatics analysis. (**A**) Matchstick analysis of the top 20 up- and downregulated metabolites in the 5 μM group. (**B**) Matchstick analysis of the top 20 up- and downregulated metabolites in the 10 μM group. (**C**) Heatmap of metabolic pathways in the 5 μM group. (**D**) Heatmap of metabolic pathways in the 10 μM group. (The higher the VIP value, the more important the metabolite is in distinguishing between different groups).

**Figure 7:**
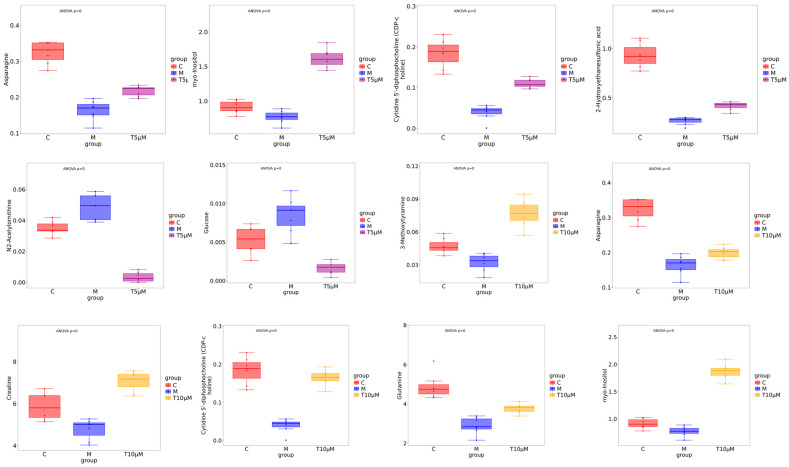
Box-plot analysis of the significantly changed metabolites in the 5 μM and 10 μM groups. (All differential metabolites were analyzed using analysis of variance, *p* < 0.00001.)

Key small molecule metabolites modulated by GATC were further selected with a fold change of ≥2 or ≤0.5. Figure 6A,B displays the fold change of the top 20 metabolites, highlighting the significant variations in DEMs and suggesting that the expression of corresponding genes may be significantly upregulated or downregulated. In the 5 T group, enriched metabolites such as CDP-choline, 2-keto-3-deoxygalactonic acid, myo-inositol, and adenylsuccinic acid exhibited significant increases compared to the M group (Figure 6A). In the 10 T group, the levels of CDP-choline, 2-keto-3-deoxygalactonic acid, myo-inositol, and taurocholic acid were increased, whereas metabolites associated with glucometabolism such as sorbose, glucose, cellobiose, and maltose were decreased (Figure 6B). Based on the enrichment analysis of DEMs within the KEGG metabolic pathways, we calculated the Rich Factor, a metric that quantifies the enrichment level of specific pathways. A higher Rich Factor value corresponds to a greater degree of enrichment, reflecting the relative significance of the metabolic pathways associated with the DEMs. The KEGG enrichment plot and heatmaps for DEMs are presented in Figure 6C,D. The relevant pathways included arginine biosynthesis, the biosynthesis of amino acids and co-factors, glucose metabolism-related pathways, GPI-anchor biosynthesis, glycerophospholipid metabolism, linoleic acid metabolism, taurine and hypotaurine metabolism, and mTOR signaling pathways in the 5 T group, together with neuroactive ligand–receptor in the 10 T group. To facilitate a visual assessment of the variation in representative metabolite levels among distinct experimental groups, we employed box-plots for graphical representation (Figure 7).

### 3.6. The Effect of GATC on the Phosphorylation of AMPK

**Figure 8:**
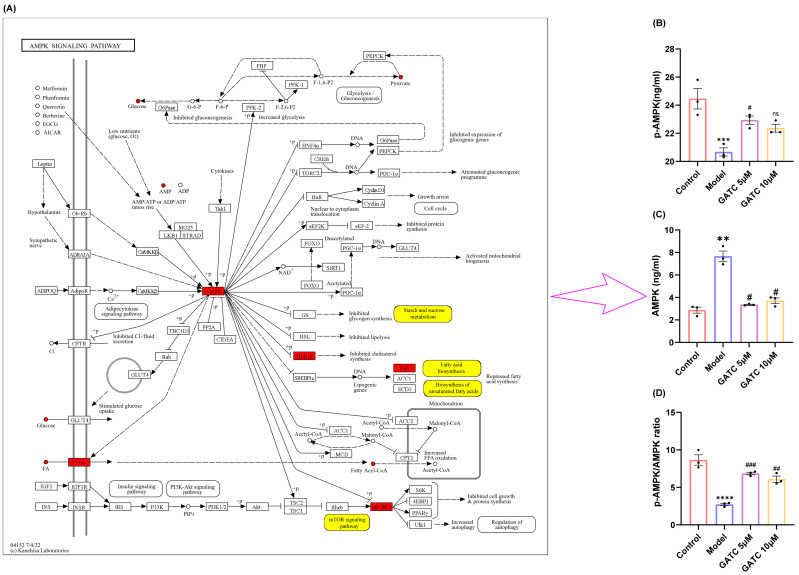
Annotation of differential metabolites and key genes in the AMPK pathway and expression of AMPK pathway-related proteins and their phosphorylated proteins in various groups of liver cells during GATC treatment. (**A**) Annotation of differentially expressed metabolites (in yellow) and key genes (in red). (**B**) AMPK expression. (**C**) p-AMPK expression. (**D**) p-AMPK/AMPK calculated value. (vs. control group **** *p* < 0.0001, *** *p* < 0.001, ** *p* < 0.01; vs. model group ### *p* < 0.001, ## *p* < 0.01, # *p* < 0.05, n = 3).

The metabolic profiling results suggest that the mechanisms of GATC may involve the AMPK pathway, which is implicated in the downregulation of mTOR, the reversion of inflammation, and the modulation of fatty acid synthesis and lipid metabolism (Figure 8A). AMPK is recognized for its pivotal role in lipid metabolism, as it regulates key enzymes and transcription factors that control lipid synthesis and breakdown. This regulation by AMPK is essential for maintaining energy homeostasis and is significant for the development of metabolic disorders. With these considerations in mind, we proceeded to design experiments to determine whether GATC alleviates HFD-induced MASLD by modulating the AMPK pathway. As a result, the phosphorylation of AMPK in the liver was significantly reduced following a period of HFD intake compared to the control group. However, in contrast to the HFD group, AMPK phosphorylation levels were significantly increased after GATC intervention, as depicted in Figure 8B–D. In summary, GATC’s protective effect is associated with activating the AMPK signaling pathway, potentially alleviating HFD-induced MASLD by modulating lipid metabolism and suppressing inflammation.

### 3.7. Interactions Between GATC and Target Proteins Using Molecular Docking

**Figure 9:**
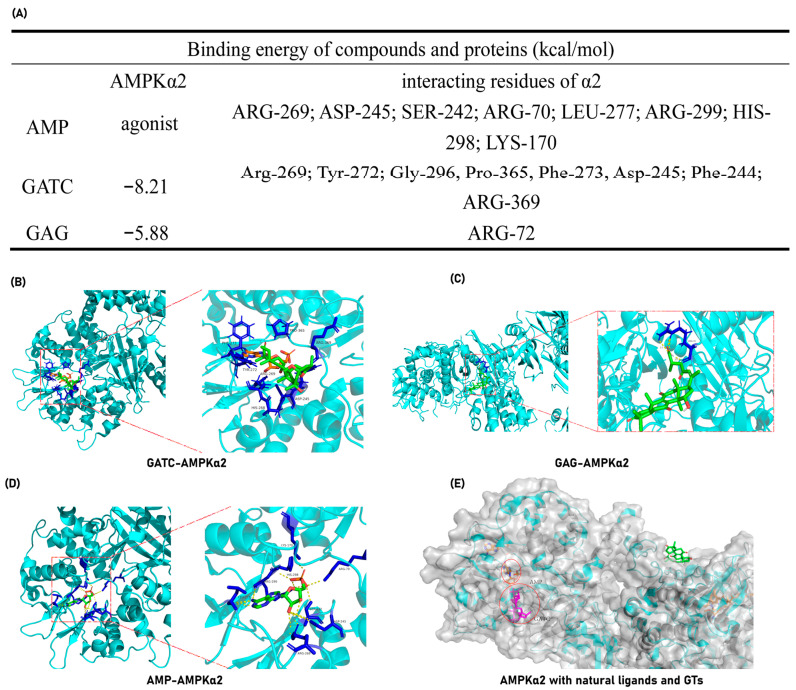
GATC and GAG docking with AMPKα-2. (**A**) Binding energy and interacting amino acid residues. (**B**) Complex of GATC and AMPKα-2; (**C**) Complex of AMP (natural agonist) and AMPKα-2; (**D**) Complex of GAG and AMPKα-2; (**E**) The surface representations of AMPK in complex with AMP, GATC, and GAG were generated and superimposed following molecular docking to facilitate a comparative analysis of their binding modes.

The experimental findings ultimately pinpointed the mechanism of action of GATC to the AMPK pathway, suggesting that GATC may function as an AMPK activator. To further validate this hypothesis, molecular docking experiments were conducted to assess the binding energy and binding sites between GATC and AMPK. Additionally, for comparative analysis, docking studies were performed with the well-characterized triterpene GAG to elucidate the structural determinants influencing the binding interactions among different triterpenes. GATC demonstrates a robust binding affinity to AMPK, characterized by a notably low binding energy of −8.21 kcal/mol (Figure 9A). Detailed analysis of the binding site indicates that the carbonyl group at C-27 of GATC forms a hydrogen bond with the 369-Arg residue. The remainder of the molecule extends into the hydrophobic pocket within the protein’s cavity, engaging in hydrophobic interactions with several key residues, including Arg-269, Tyr-272, Gly-296, Pro-365, Phe-273, Asp-245, and Phe-244 (Figure 9B). This binding site significantly overlaps with the region where AMP, the natural agonist of AMPK, binds. Among these, Arg-269 and Asp-245 are particularly noteworthy as they are shared interaction sites for both GATC and AMP, suggesting a potential mechanism of action similar to that of the endogenous ligand (Figure 9A–C,E).

In contrast, molecular docking studies of GAG revealed that it does not occupy the active cavity of AMPK (Figure 9C). Instead, GAG is positioned on the surface of the protein, indicating a lack of direct interaction with the critical binding site required for AMPK activation.

## 4. Discussion

The liver is the primary regulator of lipid homeostasis, regulating fatty acid uptake, synthesis, oxidative metabolism, and lipid distribution throughout the body [25]. Disruptions in the liver’s lipid metabolism can lead to abnormal lipid accumulation, oxidative stress, and liver damage, which are key contributors to various liver diseases, particularly MASLD and other hepatopathies [26]. In this study, activity-guided chemical investigations of G. applanatum led to the isolation of four GTs. GATA and GATC were distinctive compounds in this fungus with a unique C-23 spiro 5/7 system, while MGI and GAG represented classic lanostane-type triterpenoids widely distributed in all *Ganoderma* species (Figure 1). In vitro evaluation revealed that all four GTs exhibited lipid-accumulation activity to varying extents at a concentration of 10 μM, with GATC being the most promising, matching the lipid-clearing activity of atorvastatin (ATV). Therefore, GATC was selected for further activity testing and mechanism studies to explore its potential as the active ingredient behind the medicinal effects of *G. applanatum* in traditional remedies.

### 4.1. Lipid Metabolism and the “Two-Hit” Model in MASLD Pathogenesis

The pathogenesis of MASLD is commonly explained by the “two-hit” model [27]. The “first hit” involves increased insulin resistance from HFD intake, leading to liver adipocyte infiltration and lipid accumulation [28], with particularly pronounced accumulation in the liver, stomach, and vitelline membrane (Figure 2A,B). Such liver damage resulting from lipid accumulation is distinctly observable via H&E staining (Figure 3A). The lipid clearance rate of the compound GATC at a low concentration (2.5 μM) was comparable to the positive control ATV at 2.2 μM. The clearance rates of GATC at concentrations of 5 μM and 10 μM were ever better than ATV at 2.2 μM (Figure 2F). It has been associated with liver lipid accumulation, leading to hepatocyte membrane damage, followed by triglyceride (TG) accumulation and elevated levels of aspartate transaminase (AST) and alanine transaminase (ALT) in the liver, serving as reliable biomarkers for the assessment of MASLD [29,30,31]. This study reveals that GATC at 5 μM significantly lowers the activities of AST and ALT, along with the levels of TG, as depicted in Figure 2G and Figure 3B,C.

The “second hit” describes additional stressors on compromised liver cells, such as inflammatory cytokines and oxidative stress, which can lead to hepatocyte inflammation, necrosis, or fibrosis [31,32,33]. In this study, HFD consumption triggered the production of ROS, which diminished the activity of antioxidant enzymes, particularly SOD, CAT, and GSH-Px (Figure 4A–C). Additionally, the levels of malondialdehyde (MDA), a well-established biomarker of lipid peroxidation, were significantly elevated following high-fat diet (HFD) administration. GATC at concentrations of 5 μM and 10 μM notably elevated the levels of SOD, CAT, and GSH-Px while concurrently lowering the levels of MDA (Figure 4A–D). These results suggest that GATC has the capacity to mitigate oxidative stress and the hepatic injury induced by HFD. In addition to oxidative stress, inflammatory activation and hepatocyte apoptosis are key contributors to MASLD. During inflammatory responses, the increase in IL-6 occurs earlier than that of other cytokines, and the level of IL-6 elevation correlates with the severity and persists longer; therefore, IL-6 can be used to assist in the early diagnosis of acute infections. The upregulated IL-6 in the context of inflammatory responses, alongside the secretion of other cytokines such as TNF-α and IL-1β, collectively contribute to the complex immunological reactions in MASLD. Our investigation has elucidated the capacity of GATC to effectively downregulate the expression of IL-6, TNF-α, and IL-1β (Figure 4E–G), thereby indicating its prospective role in alleviating inflammation triggered by HFD.

### 4.2. Metabolic Reprogramming

Metabolic reprogramming is a central feature of MASLD, involving the dysregulation of glycolysis, amino acid metabolism, and lipid metabolism. To understand how GATC modulates this process in vivo, we examined metabolite variations in zebrafish using LC-MS. This technique identified the relationships between metabolic markers and pathways, providing insights into GATC’s mechanisms. Non-targeted metabolomics analysis using PCA and K-means clustering revealed significant metabolite profile alterations in zebrafish treated with GATC (5 T and 10 T) (Figure 5A,B). GATC treatment lowered blood lipids by affecting lipid-like molecules (11.49%/14.55%), benzenoids (14.94%/14.55%), and organoheterocyclic compounds (16.67%/9.86%) (Figure 5E,F). Key metabolites such as lactose, glucose, and sorbose were downregulated, while CDP-choline, myo-inositol, and glutamine were upregulated (Figure 6A,B). CDP-choline, essential for phosphatidylcholine (PC) synthesis, maintains hepatocyte membrane integrity and protects mitochondria from oxidative stress [34,35,36,37]. Myo-inositol (MI), synthesized from D-glucose, regulates cell growth, energy balance, and exhibits anti-inflammatory, antioxidant, and anti-diabetic properties, making it a potential therapeutic for MASLD [38,39,40]. Given these attributes, MI may emerge as a potent component in the therapeutic management of MASLD. In MASLD, it also has been reported that the deregulated metabolism of the amino acid glutamine is implicated, characterized by an increased necro-inflammatory response against a background of steatosis. Glutamine metabolism is deregulated in MASLD, contributing to liver injury. Its supplementation is being explored for liver damage amelioration [41]. Other metabolites such as asparagine and glucose showed distinct distributions across groups (Figure 7). Enrichment analysis of KEGG pathways revealed GATC’s significant impact on glycolysis, amino acid metabolism, lipid metabolism, taurine metabolism, and the mTOR pathway (Figure 6C,D). Elevated taurine levels in the GATC group suggest its role in modulating taurine metabolism. GATC may mitigate HFD-induced liver damage by reducing oxidative stress and inflammation.

### 4.3. The AMPK/mTOR Pathway

Previous research has identified that during the “second hit” phase in MASLD progression, the AMPK signaling pathway, functioning as a metabolic regulator that regulates energy homeostasis, is intricately implicated in the entire spectrum of MASLD’s onset and progression. Metabolic reprogramming is a central feature of MASLD, involving the dysregulation of glycolysis, amino acid metabolism, and lipid metabolism. The non-targeted metabolomics analysis revealed that GATC significantly influenced these pathways [42,43,44]. Therefore, we suspect that GATC regulates these metabolites through the MAPK pathway. The mTOR pathway, which is also regulated by the AMPK pathway, plays a pivotal role in metabolic regulation and is critically involved in the pathogenesis of MASLD. In the liver, mTORC1 activation promotes lipogenesis and inhibits autophagy, leading to lipid accumulation and hepatocyte damage. This is further exacerbated by the pathway’s role in enhancing inflammatory responses through the activation of NF-κB and the production of pro-inflammatory cytokines. AMPK activation inhibits mTORC1 through the phosphorylation of the TSC1/2 complex or the direct repression of Raptor, thereby reducing lipid synthesis and inflammation [45,46]. In this study, we assessed the levels of phosphorylated AMPK and total AMPK. AMPK phosphorylation was found to be reduced in the model (M) group compared to the control (C) group, with a significant increase observed following GATC treatment (Figure 8). In MASLD, enhancing AMPK activity can suppress the synthesis of fatty acids and cholesterol. This is achieved by reducing the expression of genes associated with adipogenesis, such as FAS, SREBP-1c, ACC, and HMGCR [33]. Currently, certain AMPK activators are considered advantageous for therapeutic interventions. Hence, the activation of the AMPK signaling pathway emerges as a promising therapeutic strategy for liver-related disorders.

Molecular docking studies reveal that GATC exhibits strong binding affinities to the target proteins AMPKα2 and AMPKβ2, which are directly related to the AMPK pathway. Furthermore, GATC exhibits strong binding affinities to CASP1, CD36, and Nrf2. Under the same conditions, one of the classic lanosterol GTs, GAG, was also subjected to molecular docking with the same proteins, but GATC has a lower binding energy (Figure 9A). The binding site analysis shows that GATC aligns with the natural active ligand binding regions of most proteins, suggesting a high degree of complementarity (Figure 9B,C). It can be clearly seen that specific structural fragments, the C-23 spiro 5/7 system, and the C-20 hydroxyl group are the key segments for GATC to bind with target proteins. Based on these findings, we hypothesize that GATC may possess anti-inflammatory properties by suppressing the inflammasome and reducing the production of inflammatory mediators. Additionally, GATC appears to activate AMPK phosphorylation and inhibit the mTOR pathway, which could contribute to a decrease in lipid accumulation and oxidative stress. The antioxidant effects of GATC may also be associated with the activation of Nrf2, a key transcription factor in the cellular response to oxidative stress. Furthermore, GATC’s interaction with the lipid transport protein CD36 suggests a potential role in impeding lipid transport and facilitating lipid clearance.

### 4.4. Clinical Implications and Future Directions

The translational relevance of our findings is underscored by the evolutionary conservation of lipid metabolic pathways between zebrafish and humans. Rodents lack cholesteryl ester transfer protein (CETP), which causes them to be resistant to the development of MASLD, making the establishment of MASLD in rodent models more costly and time-consuming. At the same time, zebrafish have 87% gene homology with humans and carry human homologous CETP [47]. Therefore, using the zebrafish model to construct the MASLD model is an efficient, rapid, and low-cost method. Meanwhile, emerging research demonstrates that AMPK exerts suppressive effects on hepatic lipid biosynthesis and sterol production while enhancing the mitochondrial β-oxidation of fatty acids. Pathophysiological studies have identified impaired AMPK signaling as a critical contributor to MASLD progression, with multiple preclinical investigations demonstrating that pharmacological AMPK activation effectively attenuates hepatic steatosis and inflammation in experimental models. These collective findings substantiate AMPK’s position as a promising therapeutic node for MASLD and its inflammatory sequelae [48]. This conclusion further supports the therapeutic potential of targeting this pathway. Although zebrafish lack some mammalian-specific transporters, our molecular docking data predict a strong binding affinity of GATC to human AMPKα (PDB: 4CFE), reinforcing its translational promise.

Our findings highlight GATC as a promising therapeutic candidate for MASLD, demonstrating significant lipid-clearing, antioxidant, and anti-inflammatory activities at concentrations of 5 µM and 10 µM. Meanwhile, compound GATC conformed to the Lipinski 5 Rules. Detailed drug-like and simulated pharmacokinetic data can be found in “Durg-like prediction of GATC” in the Supplementary File. The survival curve provided in the Supporting Information further confirms the safety of GATC with no observed toxicity over 6 days at therapeutic doses (5 µM and 10 µM). Even at a higher concentration of 80 µM, GATC exhibited only mild toxicity, which was less severe than the metabolic stress induced by a high-fat diet (Figure S1, Supporting Information). The effective concentrations of GATC, combined with its wide therapeutic window (5–80 µM), provide a robust foundation for further preclinical dose optimization. Future studies should prioritize elucidating the long-term safety and efficacy of GATC, optimizing dosing strategies, and validating its effects in mammalian models before advancing to human clinical trials. Additionally, further investigation into the molecular mechanisms underlying GATC’s effects—particularly its modulation of the AMPK/mTOR pathway—will offer critical insights for developing targeted therapies for MASLD.

## 5. Conclusions

In this study, we identified and isolated four ganoderma triterpenoids (GTs), including GATC, from the medicinal mushroom *G. applanatum*. Compound GATC is characterized by a distinctive C-23 spiro 5/7 system. Among these compounds, GATC exhibits potential multi-target therapeutic effects akin to various natural bioactive substances and is capable of regulating the metabolism of amino acids, sugars, and lipids, as well as lipid transport. Furthermore, its lipid-clearing and hepatoprotective activities indicate that GATC can mitigate inflammatory responses and oxidative damage. The hepatoprotective effects of GATC may involve the activation of the AMPK/mTOR pathway. However, the mechanism of this pathway requires further investigation. Our findings confirm that *G. applanatum* is abundant in bioactive metabolites, making it a promising subject for further research. This research could provide novel insights into the medicinal and dietary management of MASLD progression.

## Data Availability

The raw data supporting the conclusions of this article will be made available by the authors on request.

## Supplementary Materials

The following supporting information can be downloaded at: https://www.mdpi.com/article/10.3390/antiox14060637/s1. References [49,50,51,52,53,54,55,56,57] are cited in the Supplementary Material.
