# Supplementary material for: Ganoapplanilactone C from Ganoderma applanatum Ameliorates Metabolic Dysfunction-Associated Steatotic Liver Disease via AMPK/mTOR-Mediated Lipid Regulation in Zebrafish
Source: Antioxidants (Basel). 2025 May 26;14(6):637. doi: 10.3390/antiox14060637 (PMC12189419; doi:10.3390/antiox14060637)
Supplement: Supplementary file 1 [file antioxidants-14-00637-s001.zip › supporting information.pdf]

Supporting information

Ganoapplanilactone C from *Ganoderma applanatum* ameliorates metabolic dysfunction-associated steatotic liver disease via AMPK/mTOR-mediated lipid regulation in zebrafish

Yi-Fan Guo[a], Meng-Ke Zhang[a], Jia-Yang Xu[a], Xin-Ru Guo[a], Meng-Yue Dong[a], Xin Chen[a], An-An Yang[a], Jin-Ming Gao*, Xia Yin*, [a]

[a]Shaanxi Key Laboratory of Natural Products & Chemical Biology, College of Chemistry & Pharmacy, Northwest A&F University, Yangling 712100, Shaanxi, People's Republic of China

*Corresponding author (Tel: +86-29-87092335; Fax: +86-29-87092226; E-mail: yinxiabb@outlook.com (X. Yin)

1. ^{1}H and ^{13}C NMR data for Ganoapplanilactone A
2. ^{1}H and ^{13}C NMR data for Ganoapplanilactone C
3. ^{1}H and ^{13}C NMR data for Methyl gannosate I
4. ^{1}H and ^{13}C NMR data for Ganoderenic acid G
5. 18s RNA sequence of *Ganoderma applanatum*
6. Table of reagents
7. Figure S1: Larval survival under different dosing conditions.
8. Supplementary information for the non-targeted metabolomics

1. NMR data for Ganoapplanilactone A

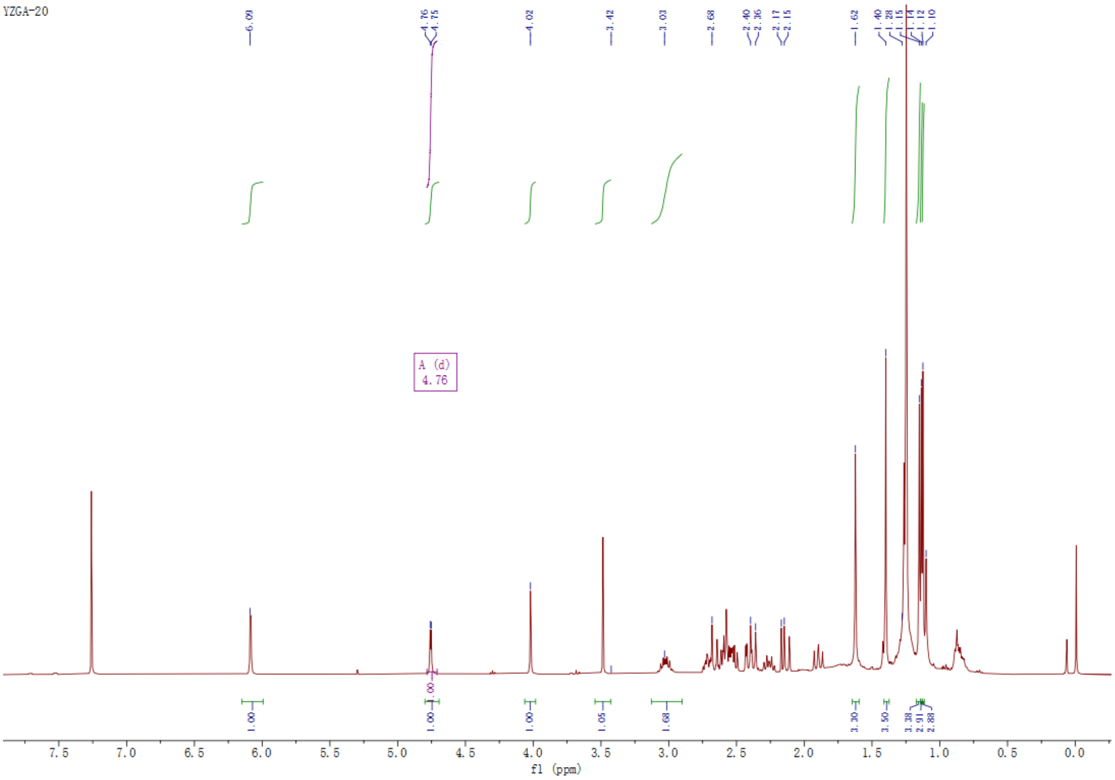

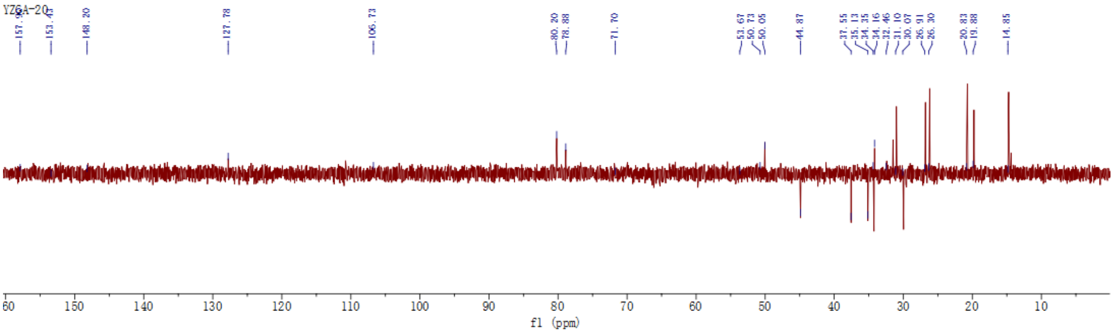

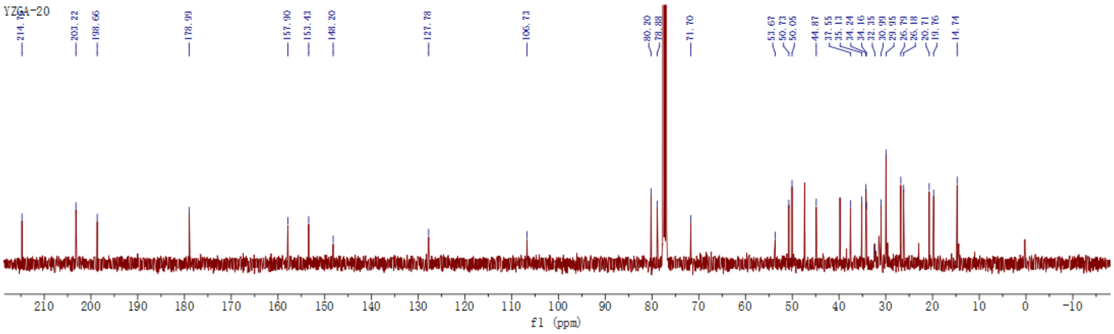

Reference

Li L, Peng XR, Dong JR, Lu SY, Zhou L, Qiu MH. 2018. Rearranged lanostane-type triterpenoids with anti-hepatic fibrosis activities from *Ganoderma applanatum*[J]. RSC Advances, 8(55): 31287-31295.

2. NMR data for Ganoapplanilactone C

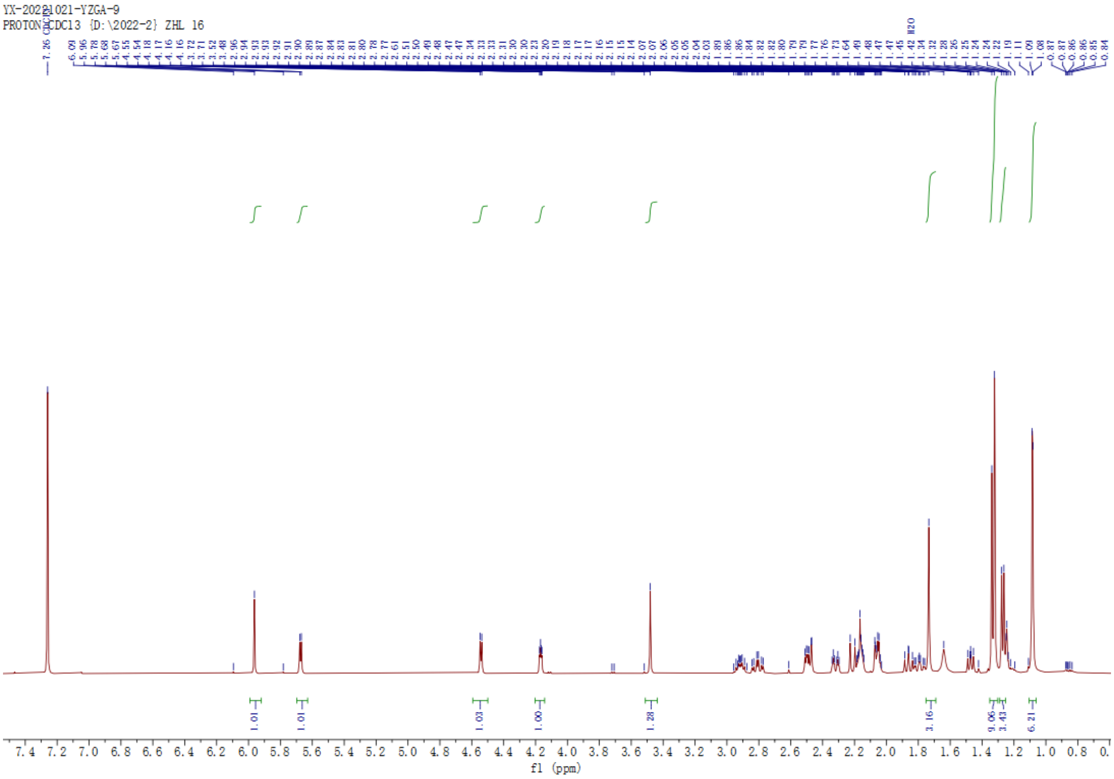

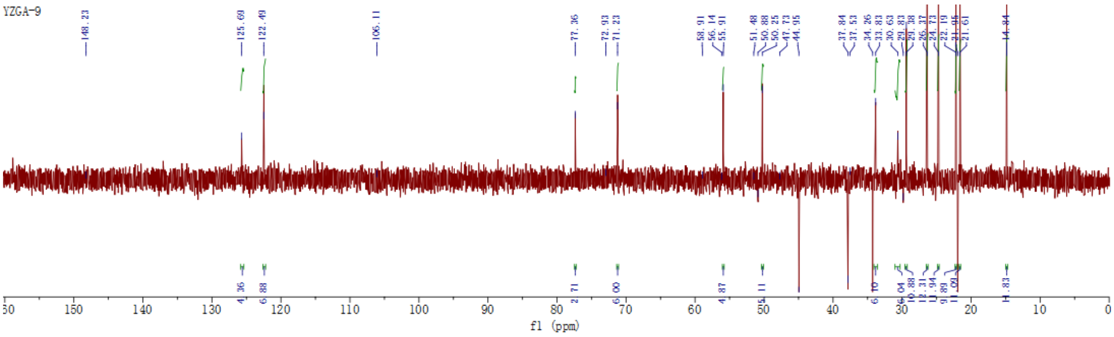

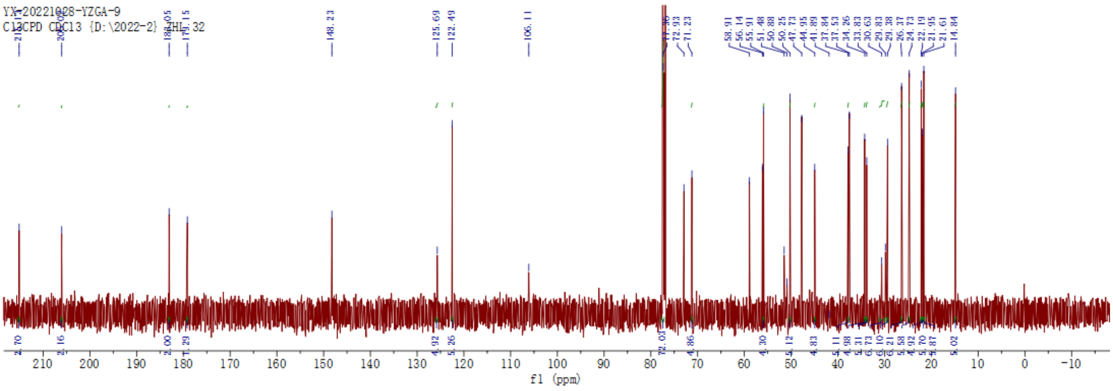

Reference

Li L, Peng XR, Dong JR, Lu SY, Zhou L, Qiu MH. 2018. Rearranged lanostane-type triterpenoids with anti-hepatic fibrosis activities from *Ganoderma applanatum*[J]. RSC Advances, 8(55): 31287-31295.

3. NMR data for Methyl gannosate I

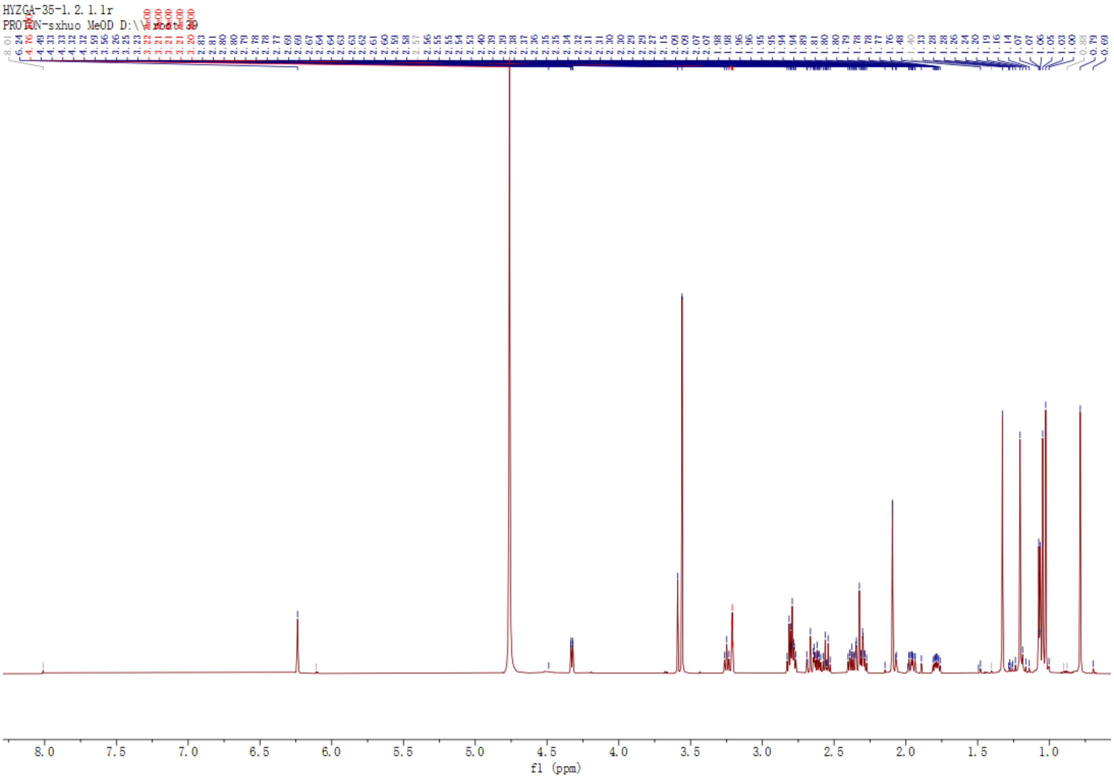

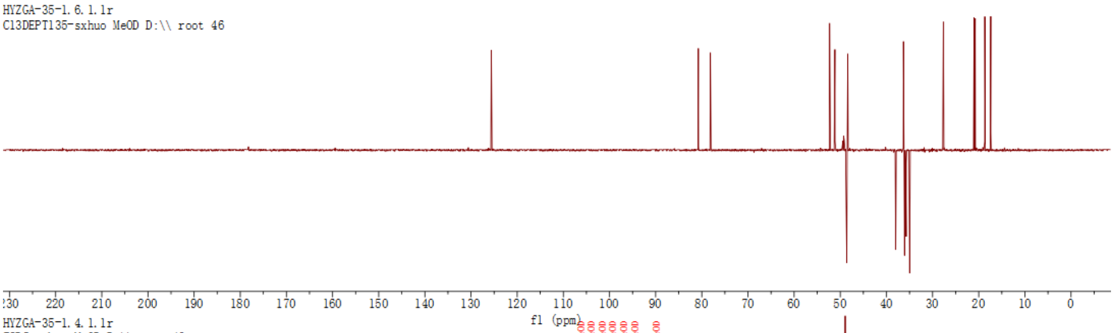

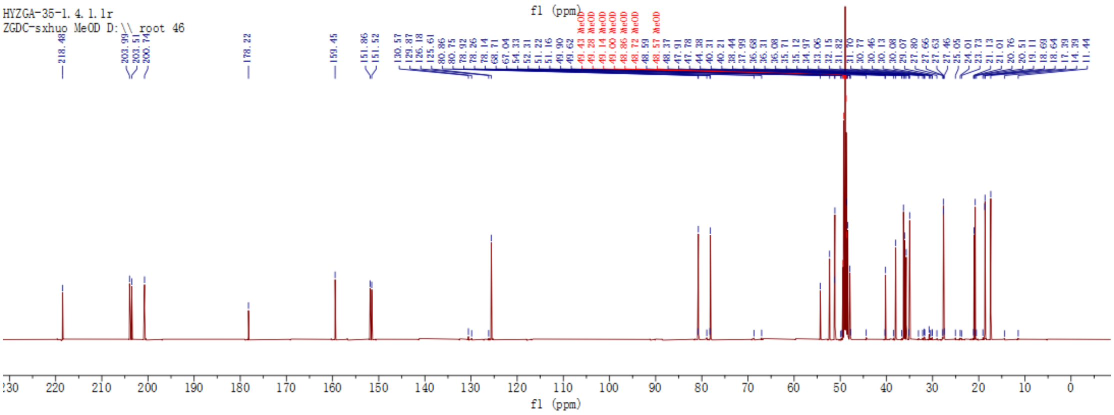

Reference

Peng XR, Wang Q, Su H, Zhou L, Xiong WY, Qiu MH. 2022. Anti-adipogenic lanostane-type triterpenoids from the edible and medicinal mushroom *Ganoderma applanatum*[J]. Journal of fungi, 8(4)

4. NMR data for Ganoderenic acid G

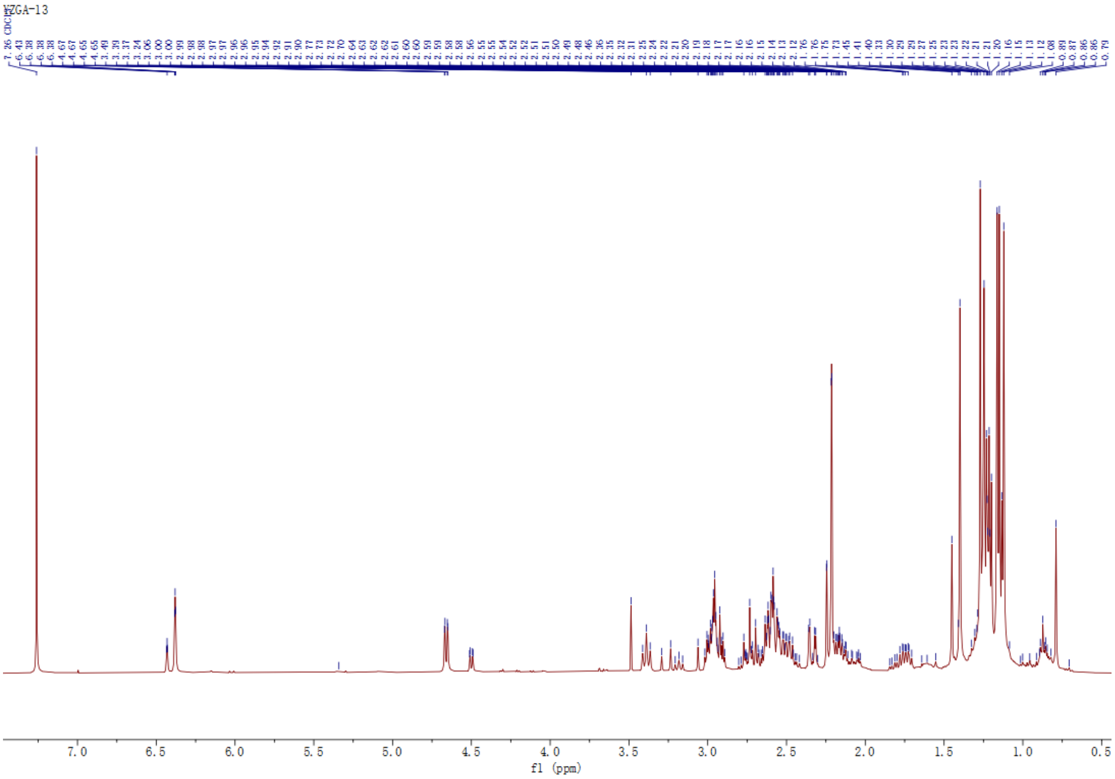

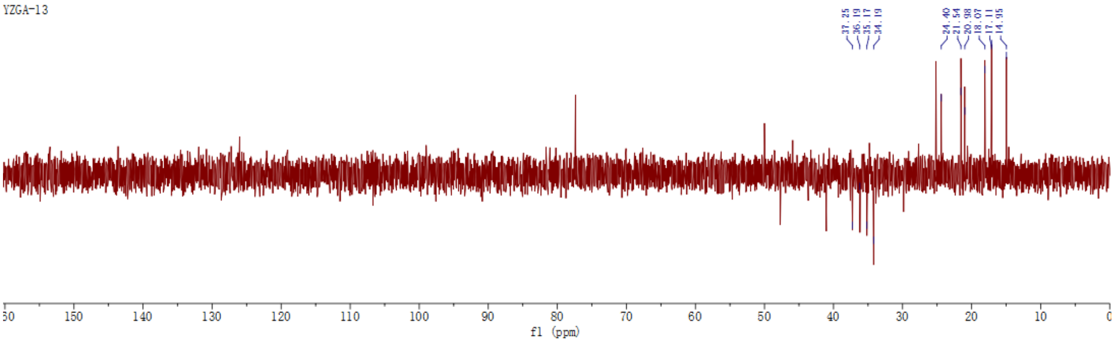

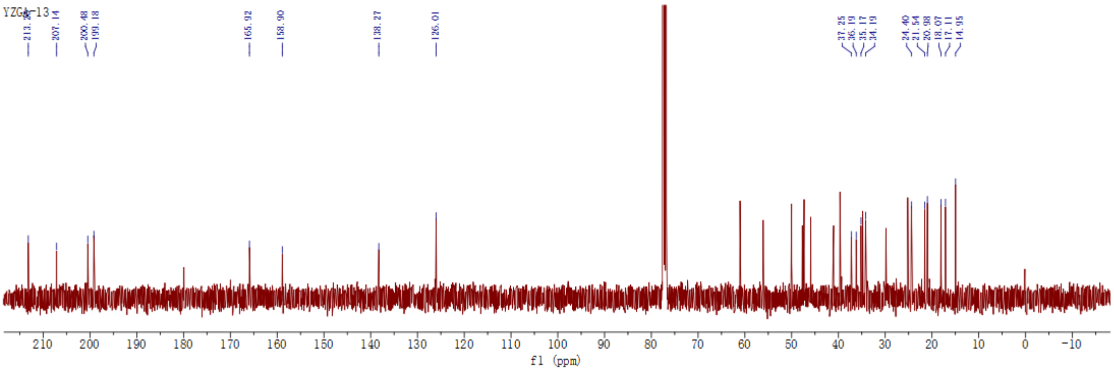

Reference

Nishitoba T, Goto S, Sato H, Sakamura S. 1989. Bitter triterpenoids from the fungus *Ganoderma applanatum*[J]. Phytochemistry, 28(1): 193-197.

5. 18s RNA sequence of *Ganoderma applanatum*

TCTACCTGATTTGAGGTCAGAGGTCATAAAAGCTGTCTCTGTAACGAGAC

GGTTAGAAGCTCGCCCAAACACGCTTCACGGTCGCGGCGTAGACATTATC

ACACCGACAAGCCGATCCGCAAGGAACCAAGCTAATGCATTTAAGAGGA

GCCGACCTGTAAAGGGACCGACAAGCCTCCAAGTCCAAGCCTACAACCCC

CATAAGAAGTTTGTAGATTGAAGATTTCATGACACTCAAACAGGCATGCT

CCTCGGAATACCAAGGAGCGCAAGGTGCGTTCAAAGATTCGATGATTCAC

TGAATTCTGCAATTCACATTACTTATCGCATTTCGCTGCGTTCTTCATCGAT

GCGAGAGCCAAGAGATCCGTTGCTGAAAGTTGTATATAGATGCGTTACAT

CGCAATACACATTCTGATACTTTATAGAGTTTGTGATAAACGCAGGAAAC

AAGTGCGCTCAACAAGCCCGAATAAACGAGCCCGTTTCACGACCCGTAAA

CCCACAGTAAGTGCACAGGTGTAGAGTGGATGAGCAGGGCGTGCACATG

CCTCGGAAGGCCAGCTACAACCCAGTCAGAACTGC

6. Table of reagents

Reagent name	Manufacturer	Catalog No.	Region
MeOH	Macklin Co., Ltd	M813907	Shanghai, China
$CHCl_3$	Macklin Co., Ltd	M102445	Shanghai, China
Acetone	Macklin Co., Ltd	H811209	Shanghai, China
Para-formaldehyde	Macklin Co., Ltd	P804536	Shanghai, China
Zebrafish	Chuangxin Biotechnology Co.	CX-A003	Hubei, China
Methanol	Xilong Science Co.	12801001	Guangdong, China
1,2-Propanediol	Xilong Science Co.	12801301	Guangdong, China
Anhydrous ethanol	Xilong Science Co.	12803401	Guangdong, China
Glacial acetic acid	Xilong Science Co.	12705701	Guangdong, China
Egg yolk powder	Yuanye Biotechnology Co.	S30910	Shanghai, China
PBS buffer	Solarbio Science & Technology Co., Ltd.	P1003	Beijing, China
Modified Oil Red O Staining Kit	Biotime Biotechnology Co.	C0158S	Shanghai, China
BCA Protein Assay Kit	Biotime Biotechnology Co.	P0010S	Shanghai, China
Total SOD Activity Assay Kit	Biotime Biotechnology Co.	S0101S	Shanghai, China
Triglyceride assay kit	Jiancheng Bioengineering Institute Co.	A110-1-1	Jiangsu, China
MDA assay kit	Jiancheng Bioengineering Institute Co.	A003-1-1	Jiangsu, China
GSH-PX assay kit	Jiancheng Bioengineering Institute Co.	A005-1-2	Jiangsu, China
CAT assay kit	Jiancheng Bioengineering Institute Co.	A007-1-1	Jiangsu, China
ALT Assay Kit	Jiancheng Bioengineering Institute Co.	C009-2-1	Jiangsu, China

AST Assay Kit	Jiancheng Bioengineering Institute Co.	C010-2-1	Jiangsu, China
p-AMPK ELISA kit	Huabang Biotechnology Co.	HB-Y10332P	Shanghai, China
AMPK ELISA Kit	Jianglai Biotechnology Co., Ltd.	JL54464	Shanghai, China
Zebrafish Interleukin 1β (IL-1β) ELISA kit	Enzyme Exemption Industry Co., Ltd.	MM-2212O2	Jiangsu, China
Zebrafish TNF-α ELISA Kit	Enzyme Exemption Industry Co., Ltd.	MM-91117O2	Jiangsu, China
Zebrafish IL6 (Interleukin 6) ELISA Kit	ELK Biotechnology Co.	ELK2419	Hubei, China

7. Figure S1: Larval survival under different dosing conditions. Data represent percent survival for n = 15 per condition (logrank test for comparing survival curves. $P < 0.001$). Day 0 represents the initiation of drug administration at 6 dpf of the larvae. The concentrations of compound GATC were 5 μM, 10 μM, 20 μM, 50 μM, and 80 μM. The concentration of HCD was 0.5%. The toxicity of GATC increased with the increase in concentration, and the survival rates of 5 μM, 10 μM, 20 μM, 50 μM, and 80 μM were 100%, 93.3%, 86.7%, 76.9%, and 66.7%, respectively, on the 6th day. The survival rate of 0.5% HCD was 53.8%.

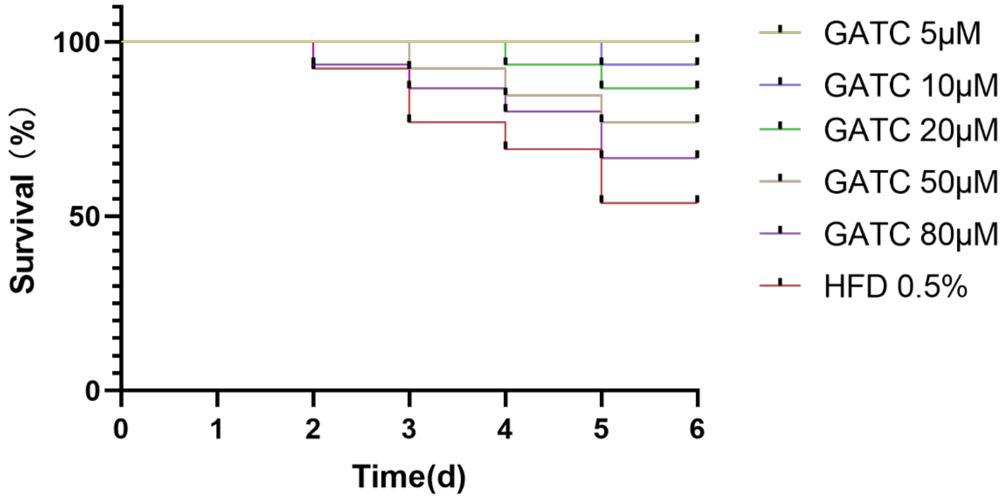

8. Supplementary information for the non-targeted metabolomics

8.1 Metabolite Extraction:

8.1.1. Metabolite Extraction from Liquids:

A total of 100 μL of sample was taken and mixed with 400 μL of extraction solution (MeOH:ACN, 1:1 (v/v)), where the extraction solution contained deuterated internal standards. The mixed solution was vortexed for 30 s, sonicated for 10 min in a 4 ℃ water bath, and incubated for 1 h at -40 ℃ to precipitate proteins. Then, the samples were centrifuged at 12000 rpm (RCF=13800(×g), R= 8.6 cm) for 15 min at 4 ℃. The supernatant was transferred to a fresh glass vial for analysis. The quality control (QC) sample was prepared by mixing an equal aliquot of the supernatant of samples.

8.1.2. Metabolite Extraction from Solids:

The animal tissue samples (25 mg±1 mg) were taken and mixed with beads and 500 μL of extraction solution (MeOH:ACN:H2O, 2:2:1 (v/v)). The extraction solution contained deuterated internal standards. The mixed solution was vortexed for 30 s. The soil samples (100 mg±1 mg) were taken and mixed with beads and 500 μL of extraction solution (MeOH:ACN:H2O, 2:2:1 (v/v)), where the extraction solution contained deuterated internal standards. The mixed solution was vortexed for 30 s. The plant samples (20 mg±1 mg) were taken and lyophilized and then mixed with beads and 1000 μL of extraction solution (MeOH:ACN:H2O, 2:2:1 (v/v)) containing deuterated internal standards. The mixed solution was vortexed for 30 s. Next, the mixed samples were homogenized (35 Hz, 4 min) and sonicated for 5 min in a 4 ℃ water bath, and this step was repeated for three times. The samples were incubated for 1 h at -40 ℃ to precipitate proteins. Then, the samples ware centrifuged at 12000 rpm (RCF=13800(×g), R= 8.6 cm) for 15 min at 4 ℃. The supernatant was transferred to a fresh glass vial for analysis. The quality control (QC) sample was prepared by mixing an equal aliquot of the supernatant of samples.

8.1.3. Metabolite Extraction from Cells:

The cell pellets (about 10^7 cells) were taken, mixed with 1000 μL of extraction solution (MeOH:ACN:H2O, 2:2:1 (v/v)), where the extraction solution contained deuterated internal standards, and the mixed solution was vortexed for 30 s and incubated in liquid nitrogen for 1 min. The samples were then allowed to thaw at room

temperature and vortexed for 30 s. This freeze–thaw cycle was repeated three times. Next, the samples were sonicated for 10 min in a 4 °C water bath and incubated for 1 h at -40 °C to precipitate proteins. The samples were centrifuged at 12000 rpm (RCF=13800(×g), R= 8.6 cm) for 15 min at 4 °C. The supernatant was transferred to a fresh glass vial for analysis. The quality control (QC) sample was prepared by mixing an equal aliquot of the supernatant of samples.

8.1.4. LC-MS/MS Analysis：

For polar metabolites, LC-MS/MS analyses were performed using a UHPLC system (Vanquish, Thermo Fisher Scientific) with a Waters ACQUITY UPLC BEH Amide (2.1 mm × 50 mm, 1.7 μm) coupled to an Orbitrap Exploris 120 mass spectrometer (Orbitrap MS, Thermo). The mobile phase consisted of 25 mmol/L ammonium acetate and 25 mmol/L ammonia hydroxide in water（pH = 9.75）(A) and acetonitrile (B). The auto-sampler temperature was 4 °C, and the injection volume was 2 μL. The Orbitrap Exploris 120 mass spectrometer was used for its ability to acquire MS/MS spectra in information-dependent acquisition (IDA) mode with the control of the acquisition software (Xcalibur, Thermo). In this mode, the acquisition software continuously evaluates the full scan MS spectrum. The ESI source conditions were set as follows: sheath gas flow rate of 50 Arb, Aux gas flow rate of 15 Arb, capillary temperature of 320 °C, full MS resolution of 60000, MS/MS resolution of 15000, collision energy: SNCE 20/30/40, and spray voltage of 3.8 kV (positive) or -3.4 kV (negative).

8.1.5. Data preprocessing and annotation：

The raw data were converted to mzXML format using ProteoWizard and processed with an in-house program, which was developed using R and based on XCMS, for peak detection, extraction, alignment, and integration. The R package and the BiotreeDB （V3.0） were applied for metabolite identification.

References:

1. Sumner LW, Amberg A, Barrett D, et al. Proposed minimum reporting standards for chemical analysis Chemical Analysis Working Group (CAWG) Metabolomics Standards Initiative (MSI). Metabolomics. 2007;3(3):211-221.

2. Dunn WB, Broadhurst D, Begley P, et al. Procedures for large-scale metabolic

profiling of serum and plasma using gas chromatography and liquid chromatography coupled to mass spectrometry. Nat Protoc 2011; 6(7): 1060-83.

3. Doppler, M.; Kluger, B.; Bueschl, C.; Schneider, C.; Krska, R.; Delcambre, S.; Hiller, K.; Lemmens, M.; Schuhmacher, R., Stable Isotope-Assisted Evaluation of Different Extraction Solvents for Untargeted Metabolomics of Plants. Int. J. Mol. Sci. 2016, 17 (7).

4. Want EJ, Wilson ID, Gika H, et al. Global metabolic profiling procedures for urine using UPLC-MS. Nat Protoc 2010; 5(6): 1005-18.

5. Yuping Cai, Kai Weng, Zheng-Jiang Zhu, et al. An integrated targeted metabolomic platform for high-throughput metabolite profiling and automated data processing. Metabolomics 2015 , 11 (6) :1575-1586.

6. Wang J, Zhang T, Shen X, et al. Serum metabolomics for early diagnosis of esophageal squamous cell carcinoma by UHPLC-QTOF/MS.Metabolomics, 2016, 12(7):116.

7. Zhou, Z., Luo, M., Zhang, H., Yin, Y., Cai, Y., & Zhu, Z. J. (2022). Metabolite annotation from knowns to unknowns through knowledge-guided multi-layer metabolic networking. Nature communications, 13(1), 6656.

8. Zhou, Z., Luo, M., Zhang, H., Yin, Y., Cai, Y., & Zhu, Z. J. (2022). Metabolite annotation from knowns to unknowns through knowledge-guided multi-layer metabolic networking. Nature communications, 13(1), 6656
